# Evaluating the Performance of Joint Angle Estimation Algorithms on an Exoskeleton Mock-Up via a Modular Testing Approach

**DOI:** 10.3390/s24175673

**Published:** 2024-08-31

**Authors:** Ryan S. Pollard, Sarah M. Bass, Mark C. Schall, Michael E. Zabala

**Affiliations:** 1 Department of Mechanical Engineering, Auburn University, Auburn, AL 36849, USA; smb0186@auburn.edu (S.M.B.); zabalme@auburn.edu (M.E.Z.); 2 Department of Industrial and Systems Engineering, Auburn University, Auburn, AL 36849, USA; mcs0084@auburn.edu

**Keywords:** exoskeleton mock-up, estimation algorithms, random forest, kinematics, single sensor, joint angles

## Abstract

A common challenge for exoskeleton control is discerning operator intent to provide seamless actuation of the device with the operator. One way to accomplish this is with joint angle estimation algorithms and multiple sensors on the human–machine system. However, the question remains of what can be accomplished with just one sensor. The objective of this study was to deploy a modular testing approach to test the performance of two joint angle estimation models—a kinematic extrapolation algorithm and a Random Forest machine learning algorithm—when each was informed solely with kinematic gait data from a single potentiometer on an ankle exoskeleton mock-up. This study demonstrates (i) the feasibility of implementing a modular approach to exoskeleton mock-up evaluation to promote continuity between testing configurations and (ii) that a Random Forest algorithm yielded lower realized errors of estimated joint angles and a decreased actuation time than the kinematic model when deployed on the physical device.

## 1. Introduction

Augmentative exoskeletons are designed to assist an operator by decreasing the effort required of a user to perform a task. This decrease in exerted effort is often characterized as a reduction in metabolic cost, muscle activity, or cognitive load [1]. Effort optimization is a function of synchronization (both in force and timing) between the operator and the wearable device [2]. Sawicki and Ferris (2017) [3] showed that merely increasing ankle joint mechanical power via an assistive exoskeleton is not directly proportional to a net metabolic power decrease, thus demonstrating that appropriate actuation timing (and not just an arbitrarily applied external joint torque) is necessary to promote fluidity between the user and robot. Several studies later explored the effect of exoskeleton actuation timing on the operator’s metabolic cost [4,5,6], each showing that the timing of joint torque application is critical in reducing metabolic cost. Additionally, operators have shown task-specific preferences for the timing and magnitude of exoskeleton actuation [7].

Each of these exoskeletons implements a cyclic torque pattern at the joint of interest, thus mitigating the controller’s relevance in transitional movements. Due to the limitations of this approach, understanding and modeling the underlying kinetics and kinematics of the human body is becoming critical to properly develop the desired trajectories of lower-limb assistive devices in online applications. Currently, two primary methods of estimating real-time human dynamics are commonly deployed: joint torque estimation and joint angle estimation [8]. Joint torques are often estimated using onboard motor currents [9], joint kinematics [10,11,12], and ground reaction forces [13], while joint angles have previously been estimated using kinematic joint data [14,15], electromyographic signals [16,17], inertial measurement units [18,19], or a combination of each [20]. Although both approaches are common throughout the existing literature on human movement prediction, the joint angle estimation approach more heavily favors synchronizing the motion of the human–robot system. This approach allows an exoskeleton’s controller to align the assistive device’s kinematic trajectory with the human operator rather than simply prioritizing the timing and profile of the applied joint torques. The performance of these joint angle estimation algorithms is often analyzed in one of two ways: post hoc evaluation using recorded gait kinematics [15,21] or deployment on a human participant via lab-based exoskeleton intervention [22,23]. However, both of these testing approaches pose significant limitations. A post hoc, offline analysis does not allow for a model’s computational performance to be realized on a physical system. Conversely, the need to test human subjects can limit an exoskeleton emulator approach by being both time- and labor-intensive.

The variability in sensor configurations, model types, control algorithms, and evaluation metrics throughout the joint angle estimation literature often causes difficulty in effectively comparing model performance for a specific application. As such, the primary aim of this study was to establish a modular approach to testing and characterizing the physical manifestation accuracy and delay of joint angle estimation models on a lower-limb exoskeleton mock-up. As the field of using non-intervention, exoskeleton mock-ups to test estimation models and control algorithms begins to expand, establishing a consistent and reliable framework for the testing process and performance metric evaluation is critical for clear communication across the literature. Such a modular approach would allow for a decrease in the expended time and effort required to test unique combinations of specific system components (such as sensor count, model type, controller design, etc.) while preserving continuity between test configurations, which would allow for a justifiable comparison of component performances. Additionally, an exoskeleton non-intervention mock-up approach would allow for the physical manifestation performance of an estimation algorithm to be evaluated without needing to deploy the prototype on a human subject. The exoskeleton mock-up presented in this study is not intended to be worn by a human subject but rather serves as an intermediate step between a purely computational joint angle estimation analysis and a physical deployment of an exoskeleton system on a human participant.

While previous research has been conducted on the accuracy of joint angle estimations calculated using a variety of sensor counts and configurations for exoskeleton applications [21], the question of “how accurate can an estimation model get while being informed by a single sensor” remains a notable gap in the scientific exoskeleton literature. While substantial literature exists on real-time pose and joint angle estimation using a reduced sensor count on the back and lower limbs [24,25,26], limited research has examined estimating joint angles with only a single sensor for exoskeleton applications. Furthermore, the studies that have been conducted have been primarily intended for rehabilitation monitoring applications and have not yet been used to estimate future joint angles [27,28,29]. Argent et al. (2019) and Alemayoh et al. (2023) deployed machine learning approaches to monitor lower limb joint angles using a single IMU sensor during online applications [27,29]. While there was brief discussion about the possibility of extending this work to inform an assistive device, neither study developed models to estimate future joint angles. However, to the knowledge of the authors, the effect of using a single potentiometer on the ankle to estimate future joint angles has not yet been explored.

There is a great deal of potential in single-potentiometer-based approaches. Pollard et al. (2024) [30] demonstrated that future joint angle estimation could be performed using simple, kinematically governed analytical models. These models can be informed solely by current and historical ankle joint angles (a parameter that can be reported using a single potentiometer or encoder at the ankle). These kinematically informed models reported significantly faster runtimes when compared to Random Forest (RF) machine learning models. However, they yielded higher estimation errors than the RF models across larger estimation horizons. As such, the second aim of this study was to deploy the aforementioned modular approach and characterize the physical manifestation accuracy and delay of two joint angle estimation models—an RF machine learning model and an analytical, kinematics-based model—informed solely by sagittal-plane ankle angle kinematics derived from a single sensor. These simple, single-sensor models will be able to serve as baseline comparators for other joint angle estimators tested within this modular configuration, thus demonstrating the baseline efficacy of simple estimation models deployed in a physical application while using a limited sensor array.

Because the RF model has previously been shown to demonstrate a lower offline estimation error compared to the kinematically informed model [30], we hypothesized that the RF model would likewise demonstrate a smaller realized estimation error on the physical exoskeleton mock-up system when compared to the kinematic extrapolation algorithm. We also hypothesized that the kinematic extrapolation algorithm would demonstrate a shorter delay in realizing the estimated joint angles in the mock-up testbed than the RF model.

## 2. Materials and Methods

### 2.1. Testing Modules

**Figure 1:**
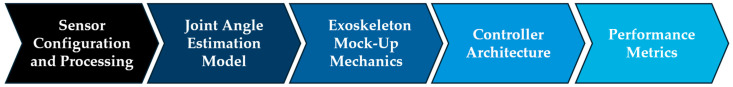
An outline of the modular approach to joint angle estimation and exoskeleton mock-up testing. Each module can be replaced or modified independently when evaluating alternative testing configurations.

The proposed testing approach seeks to modularize the exoskeleton mock-up testbed, allowing any “exchangeable” or “modifiable” component to be categorized into one of five distinct categories: sensor configuration, the joint angle estimation model, exoskeleton mock-up mechanics, controller architecture, and performance metrics, as seen in Figure 1. Each of the five modules is described in detail below.

**Figure 2:**
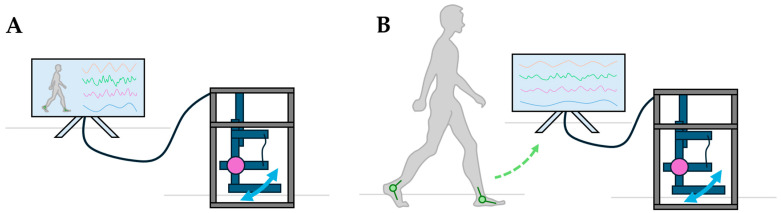
An illustration of the two methods by which an exoskeleton mock-up can be informed via arbitrary sensors and a detached operator. (**A**) Pre-recorded kinematic/kinetic data can be used to conduct an offline performance analysis of the testbed. (**B**) Human subject kinematic/kinetic data can be recorded simultaneously as the mock-up is being tested in an online performance analysis of the testbed. Both the sensor on the exoskeleton mock-up (in purple) and the sensors on the human subject (in green) are accounted for in the sensor configuration and processing module. As this study explores the effect of using a single sensor to inform an exoskeleton mock-up (Section 2.3*)*, a limited sensor count is illustrated above.

1. The sensor configuration and processing module refers to all aspects of sensor selection, count, placement, and filtering. This module describes both the sensor array onboard the exoskeleton mock-up and the sensors used to collect human kinematic and/or kinetic data (which will serve as an input to the joint angle estimation module). This human subject data can either be pre-recorded or collected in real time as the exoskeleton mock-up is being tested (as seen in Figure 2). Both configurations mitigate the complexities of deploying the mechanical prototype on a human subject.
2. The joint angle estimation model module encompasses all processes of converting filtered sensor data into an estimated joint angle, regardless of the approach taken (i.e., statistical, analytical, machine learning, model-based simulation, etc.). A predictive joint angle estimation model seeks to estimate a joint’s future position, rather than estimating the joint’s current position, so that an exoskeleton has sufficient time to actuate alongside the operator (rather than lagging behind the operator’s intended motion).
3. The exoskeleton mock-up mechanics module refers to both the structural design and the actuation method of the physical mock-up.
4. The controller architecture module describes the process by which the exoskeleton mock-up is controlled to actuate to a desired, estimated joint angle.
5. The performance metrics module is the broadest category and does not have a direct impact on the behavior of the test setup. However, this module has been included to provide further clarity when comparing different systems. Just within a single review on kinematic estimation and prediction models [31], at least five unique metrics were used to characterize the performance of the reviewed models (including mean absolute error, mean squared error, mean relative error, root-mean-squared error, and normalized root-mean-squared error), causing comparisons between model types to become more convoluted than simply comparing two numbers. Additionally, deploying joint angle estimation models on a physical system may require additional metrics to fully characterize the performance, such as computational delays and the model’s tendencies to lead or lag behind the desired joint angle estimations.

This modular approach was used as the framework for assembling an exoskeleton mock-up testbed and deploying and evaluating two different joint angle estimation models.

### 2.2. Participants

Twenty healthy individuals (9 males, 11 females; age: 22.6 ± 4.3 years; height: 173.1 ± 10.2 cm; body mass: 69.2 ± 11.7 kg) participated in an experiment performed in the Auburn University Biomechanical Engineering (AUBE) Laboratory. The data recorded for each participant would later be used to conduct an offline performance analysis of the mock-up testbed, as outlined in Figure 2A. Willing participants provided informed consent before participating in the study, as approved by the Auburn University Institutional Review Board (no. 17-096 MR 1705).

### 2.3. Sensor Configuration and Processing

Each participant was instrumented with 79 retroreflective motion capture markers, following the point cluster technique developed and outlined by Andriacchi et al. (1998) [32]. Motion capture data were collected using a 10-camera Vicon system and Nexus software (Version 2.6.1; Vicon Motion Systems Ltd., Oxford Industrial Park, Oxford, UK) while subjects walked on a single belt treadmill for 30 s. Participants were asked to set their self-selected walking speed on the treadmill at a comfortable pace that could be maintained for the duration of the 30 s walking trial. Otherwise, participants were provided with minimal instructions and feedback during the walking trial to elicit natural gait patterns. After data collection was completed, motion capture data were post-processed in Visual3D (C-Motion Inc., Germantown, MD, USA) using a 6 Hz Butterworth filter. Subsequently, the sagittal-plane ankle angles of each participant’s walking trials were extracted after modeling the ankle joint as having three degrees of freedom without consideration for translational movement [33].

To continue to investigate the effects of including only a single sensor on the exoskeleton mock-up, an absolute magnetic encoder (as in [34]) was selected to be on board the testbed. This potentiometer was integrated into the testbed hardware (discussed in detail in Section 2.5) and measured the exoskeleton mock-up’s sagittal-plane ankle angle in real time (whereas the pre-recorded motion-capture-based data would represent the operator’s underlying gait kinematics). The potential to align these two kinematic trajectories is the primary advantage of deploying a joint-angle-estimation-based model.

### 2.4. Joint Angle Estimation Models

Two joint angle estimation models, a kinematically governed extrapolation model and an RF machine learning-based model, were deployed to predict joint angles at an estimation horizon $t_{hzn}=$ 100 ms into the future, a measure that has been commonly deployed in the literature in future joint angle estimation for exoskeleton applications [15,21,30,35]. This estimation horizon length was selected to ensure that the models would remain *predictive* in nature, even after necessary computational estimation delays ($t_{est}$) and actuation times. Both estimation models were informed solely by the three most recently recorded operator ankle angles at a given time (denoted $\theta_{i},\;\theta_{i-1},\;$and $\theta_{i-2}$). The following two subsections describe how each model developed future joint angle estimations.

#### 2.4.1. Kinematically Governed Extrapolation Model

Based on a simplified version of the average angular acceleration model by Pollard et al. (2024) [30], the kinematically governed extrapolation model is defined by the analytical, constant acceleration kinematic equation, given by

(1) $$\hat{\theta }=\theta_{i}+\omega_{i}t_{hzn}+\frac{1}{2}\alpha_{i}{t_{hzn}}^{2},$$

where $\omega_{i}$ is the current average angular velocity and $\alpha_{i}$ is the current average angular acceleration, each given by

(2) $$\omega_{i}=\frac{\theta_{i}-\theta_{i-1}}{{\Delta t}_{i}},$$

and…

(3) $$\alpha_{i}=\frac{\omega_{i}-\omega_{i-1}}{{\Delta t}_{i}}=\frac{\theta_{i}-{2\theta }_{i-1}+\theta_{i-2}}{{{\Delta t}_{i}}^{2}},$$

where ${\Delta t}_{i}$ is the period of the recorded data (i.e., the time elapsed between recorded joint angle datapoints). As is evident from these equations, the three most recently recorded operator ankle angles are numerically differentiated to develop average angular velocities and accelerations for each instance. The future joint angle is estimated by assuming that the angular acceleration will remain constant over the estimation horizon.

Additionally, to prevent numerical differentiation from yielding unrealistic or exaggerated kinematic quantities, the average angular velocity and angular acceleration were constrained to physically feasible kinematic values for level ground and healthy gait (i.e., $\omega \in \left[-296.9,\;247.7\right]$ deg/s and $\alpha \in \left[-7059.6,\;4336.5\right]$ deg/s/s) [36]. This constraint does not completely correct for the error introduced by numerically differentiating discrete data points, but it does seek to mitigate the effect of extreme outliers.

#### 2.4.2. Random Forest Machine Learning Model

The second model developed in this study was a subject-independent RF machine learning model. An RF regression algorithm develops estimations based on the majority performance of a collection of individual binary decision trees. A subject-independent model structure was selected so that a singular, governing model could be deployed to estimate joint angles, similar to the kinematic extrapolation model (rather than developing a unique, subject-dependent model for each participant, as in [30]). The RF estimation model was developed using the scikit-learn Python library with $n_{estimators}$ = 5 and depth = 10.

The RF machine learning model was trained on the sagittal-plane ankle angles of 10 randomized participants’ gait trials. The model was trained by relating a sliding window of the three most recent joint angle data points (a 1 × 3 vector $\left[\theta_{i-2},{\;\theta }_{i-1},\;\theta_{i}\;\right]$) to a participant’s actual joint angle at $t_{hzn}=$ 100 ms into the future. After training, the data from these 10 randomized participants were set aside to ensure that only unseen data would be used to test the model when deployed in the modular test configuration.

### 2.5. Exoskeleton Mock-Up Mechanics

**Figure 3:**
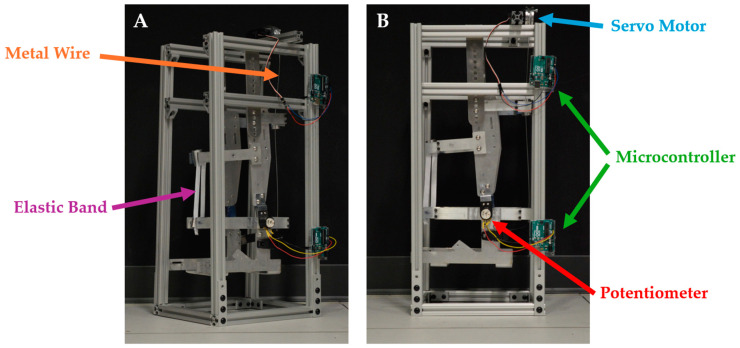
(**A**) Oblique view and (**B**) side view of the ankle exoskeleton mock-up and frame, based on [34]. The two Arduino Uno microcontrollers (green) on the frame allowed for communication between the singular potentiometer on the ankle (red), the servo motor (blue), and computer-based test software (not shown). The posterior metal cable (orange) and anterior elastic band (purple) aided in plantarflexion and dorsiflexion of the ankle joint, respectively.

A detached (no operator), aluminum-frame ankle exoskeleton mock-up was adapted and manufactured based on an open-source design developed by Bryan et al. (2020) [34] and was suspended within an aluminum extrusion t-slot frame, as shown in Figure 3. The exoskeleton mock-up was designed to have one degree of freedom in the sagittal plane about its representative ankle joint, allowing for both plantarflexion and dorsiflexion about the ankle joint (measured via a singular potentiometer; Section 2.3). A metal cable was routed from a 5 V Hitec continuous-rotation servo motor on the superior aspect of the mock-up to the effective “heel” on the inferior posterior aspect of the mock-up. This cable passed through two rotating conduits on the posterior aspect of the mock-up, aiding in active plantarflexion by functioning as a representative gastrocnemius (calf muscle) and Achilles tendon. An elastic band (measured $k_{spring}$ = 44.7 ± 3.5 N/m) was affixed to the front of the mock-up to assist with passive dorsiflexion. Two Arduino Uno microcontrollers, located on the exoskeleton mock-up’s frame, were used to communicate between the potentiometer, servo motor, and computer-based software (which included both the joint angle estimation models and the controller) developed within the Robot Operating Software ROS2 (VMWare Workstation 16 Pro, Ubuntu 20). The Arduino Unos communicated with the ROS-based models and controller via serial communication at 9600 Bd.

### 2.6. Controller Architecture

Once the estimation model develops a future joint angle estimation, that estimation is immediately communicated to the exoskeleton mock-up’s controller. Subsequently, the ROS-based controller communicates with the continuous motor to directly induce plantarflexion (by increasing the tension in the metal cable) or to indirectly induce dorsiflexion (by decreasing the tension in the metal cable and allowing the elastic band to passively unstretch). Position control of the motor would be a simple solution, since a desired future joint angle position was just estimated. However, a continuous servo motor is agnostic to its absolute rotational position, making this approach unfeasible.

A proportional velocity controller, therefore, was defined to control the servo motor deployed on this exoskeleton mock-up. When the servo motor is controlled, it must be informed by a single value (ranging from 0° to 180°) that dictates both the speed and direction of the motor (0° = rotating the motor counterclockwise at full speed, 180° = rotating the motor clockwise at full speed, and 90° = no rotation), rather than being informed by a desired angular position. As such, a relationship needed to be defined between the allowable motor input values and the exoskeleton mock-up’s angular positional error, governed by…

(4) $$e=\hat{\theta }-\theta_{mockup},$$

where $\hat{\theta }$ is the desired future joint angle estimation and $\theta_{mockup}$ is the current exoskeleton mock-up angle.

**Figure 4:**
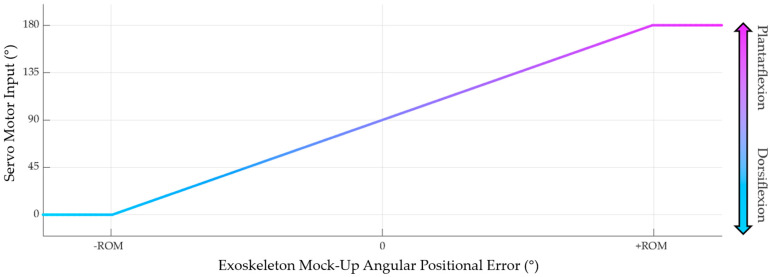
Pictorial representation of the linear mapping between the exoskeleton mock-up’s angular positional error and the servo motor’s input value. A positive error correlates to a clockwise rotation of the servo motor (active plantarflexion via tension in the metal wire). In contrast, a negative error correlates to a counterclockwise rotation (passive dorsiflexion via shortening of the elastic band).

Firstly, the minimum and maximum bounds of the positional error needed to be evaluated to allow the angular positional errors and the available motor input values to be mapped to one another. These bounds were defined as the functional range of motion (ROM) for each participant during their walking trial, given by

(5) $$ROM=\theta_{max,dorsiflexion}-\theta_{min,plantarflexion},$$

where $\theta_{max,dorsiflexion}$ is the maximum ankle angle achieved by the participant during the walking trial (occuring during peak dorsiflexion) and $\theta_{min,plantarflexion}$ is the minimum ankle angle achieved (occuring during peak plantarflexion). Analyzing a worst-case scenario, if the mock-up’s angular positional error is +ROM° (i.e., the mock-up is currently maximally dorsiflexed but desires to be maximally plantarflexed), the servo motor should quickly plantarflex the joint to correct for this error (and vice versa if the angular positional error is −ROM°). These worst-case angular positional errors can then be mapped to the maximum velocities of the servo motor, assuming a linear relationship between the extremes, as shown in Figure 4 below.

Although a proportional controller gain was not explicitly stated, the slope of the relationship depicted in Figure 4 demonstrates the “effective” controller gain that was used in this study. This controller gain was established on a subject-specific basis due to the variability between participant ROMs. If an angular positional error greater than the participant’s range of motion were to be calculated, such as e > +ROM° or e < −ROM° (potentially because of an erroneous joint angle estimation value), the mapped value would simply be mapped as the maximum or minimum allowable servo motor input. This manually established, piecewise linear relationship between the positional error and the servo motor input describes the simple proportional velocity controller by which the exoskeleton mock-up of this study was governed.

### 2.7. Deploying the Joint Angle Estimation Models on the Mock-Up Testbed

**Figure 5:**
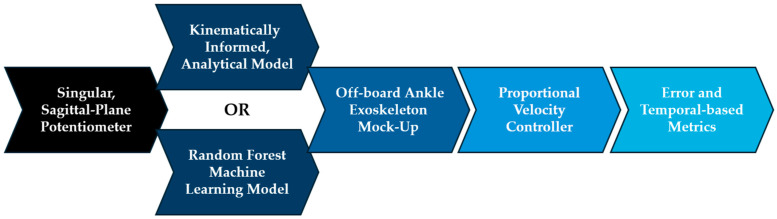
The physical manifestation performance of two joint angle estimation models (a kinematically informed analytical model and an RF machine learning model) was evaluated by deploying the modular testing approach on an exoskeleton mock-up testbed. Besides the model type, other modular parameters remained the same between tests.

After each of these modules were fully defined, two modular configurations were deployed to evaluate the performance of an exoskeleton mock-up testbed informed by either a kinematically governed model or an RF model (see Figure 5 below).

The joint angle estimation models and exoskeleton mock-up testbed were tested on the sagittal-plane ankle kinematics of the remaining 10 participants that were not used to train the RF model. For each test participant, the models’ estimated ankle angles and the exoskeleton mock-up’s realized ankle angles were recorded as the controlled system attempted to track the participants’ future ankle kinematics. The “actual” participant ankle angle, the models’ estimated joint angle at an estimation horizon $t_{hzn}=$ 100 ms into the future, and the current angular position of the exoskeleton mock-up were all published simultaneously in ROS2 during data collection. The recorded testbed data were analyzed using the following performance metrics.

### 2.8. Performance Metrics

To characterize the performance of the emulator mock-up system, a benefit may arise from defining a few different metrics to quantify various aspects of the system’s time response and the magnitude of the positional error. While there are several different methods commonly used to characterize model performance, five distinct metrics were used in this study, each of which will be defined here, and their benefits and limitations will be thoroughly discussed throughout the remainder of this article. Due to the variability in previous reporting measures [31], presenting these five metrics as the standard when reporting exoskeleton mock-up performance will allow the field to coalesce around a defined set of metrics and promote consistency across the literature. These five metrics are as follows: the model error, the realized error, the actuation time, the phase delay, and the hypothetical no-lag error. Each error metric below represents the error between specific testbed output angles and the participants’ “ground truth” joint angles recorded using optical motion capture, and each temporal metric represents a delay associated with the estimation and actuation of the exoskeleton testbed.

#### 2.8.1. Model Error

The model error describes the average error between a joint angle estimation model’s estimated future joint angles ($\hat{\theta }$) and the actual future joint angles realized by the participant during data collection ($\theta_{actual}$) that are temporally synonymous with the estimation horizon. This offline error was evaluated for both the kinematically governed model (${RMSE}_{K,model}$) and the RF model (${RMSE}_{RF,model}$).

#### 2.8.2. Realized Error

The realized error seeks to describe the average error between the mock-up’s realized joint angles ($\theta_{mockup}$) and the actual future joint angles realized by the participant during data collection ($\theta_{actual}$) that are temporally synonymous with the estimation horizon. This metric represents the compounded error of the joint model/mock-up system, characterizing how erroneous the actualized joint angles on the exoskeleton mock-up are compared to the actual kinematics of the participants. This positional error was again evaluated for both the kinematic extrapolation model (${RMSE}_{K,realized}$) and the RF model (${RMSE}_{RF,realized}$). While the realized error metric describes how erroneous the exoskeleton mock-up’s realized angular positions were relative to the underlying human kinematics, the metric does not differentiate whether the mock-up’s realized positions were incorrect altogether or if the shape of their trajectory was accurate yet simply temporally lagging behind the human’s trajectory. This inability to quantify the *direction* of the error (temporal error vs. magnitude error) is a significant limitation of the root-mean-square error metric that will be addressed with the inclusion of the following three performance metrics.

#### 2.8.3. Actuation Time

The actuation time ($t_{actuate}$) represents how long the exoskeleton mock-up takes to actuate to a desired angular position once an estimation model makes a future joint angle estimation. Feedback error is introduced throughout the actuation process due to factors such as having only a singular positional sensor to represent the current position or using a passive elastic band to induce dorsiflexion. This feedback error causes the exoskeleton mock-up angular position curve to both temporally lag behind the desired estimated joint angle curve (because of the time required to actuate the device) and erroneously misshapen (because of the feedback error when trying to actuate to the desired angle). This creates difficulty in defining the actuation time of the system, as the estimated ankle angle curve and the realized mock-up ankle angle curve are not simply similar curves with a lagged phase shift between them.

As such, a cross-correlation technique was deployed to estimate the phase shift between the joint angle estimation angles ($\hat{\theta }$) and the realized mock-up ankle angles ($\theta_{mockup}$) (similar to phase shift estimators discussed by Stoica et al. [37]). The cross-correlation between the two curves was documented as they incrementally shifted past one another. The actuation time was thus estimated as the phase shift required to maximize the correlation between the joint angle estimation curve and the realized mock-up angle curve.

#### 2.8.4. Phase Delay

Ideally, the process of estimating future joint angles and subsequently actuating to those desired angles would precede actual human motion. However, as there is often great difficulty in completing these control processes quickly enough, the phase delay ($t_{phase}$) is representative of how lagged the realized mock-up joint angles are behind the actual human kinematics. The phase delay was calculated similarly to the actuation time: by estimating the phase delay as the phase shift required to maximally correlate the actual joint angles realized by the participant during data collection ($\theta_{actual}$) and the mock-up’s realized joint angles ($\theta_{mockup}$).

#### 2.8.5. No-Lag Error

Now that the phase delays have been defined, one last error metric can be defined: the no-lag error. The no-lag error is an error metric that characterizes the magnitude of the positional error of the realized mock-up angles by assuming that the error associated with the temporal delay was minimized. This metric is important in characterizing the “best-case scenario” positional error for the mock-up when informed by different model types. Therefore, the no-lag error of the exoskeleton mock-up, informed by both the kinematic (${RMSE}_{K,nolag}$) and RF model (${RMSE}_{RF,nolag}$), was calculated to characterize the unbiased shape of the mock-up’s realized angular position with respect to the underlying human kinematic trajectory. The no-lag error was calculated by evaluating the root-mean-square error between the exoskeleton mock-up’s realized angles and the actual joint angles realized by the participant when the two trajectories were maximally correlated.

#### 2.8.6. Statistical Analysis

Each of the performance metric datasets was first tested for normality using the Shapiro–Wilk test. Upon determining that each was normally distributed, post hoc paired *t*-tests were performed to compare the effect of model type on the error and temporal performance metrics, as follows:

- One-tailed paired *t*-tests were performed to test for directional differences between joint angle estimation model types in terms of model errors, realized errors, and actuation times.
- Two-tailed paired *t*-tests were performed to test for bidirectional differences between joint angle estimation model types in terms of phase delays and no-lag errors.

A Type I error rate of α = 0.05 was used when performing each of the statistical tests.

## 3. Results

### 3.1. Performance Metric Evaluation

**Figure 6:**
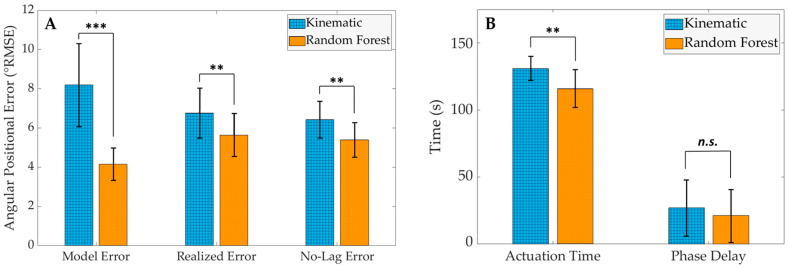
The results of the five performance metrics are grouped by (**A**) error metrics and (**B**) temporal metrics. (Results of post hoc paired *t*-tests denoted by ** *p* < 0.01; *** *p* < 0.001; n.s. = no significance).

Figure 6 provides the results of the post hoc paired *t*-tests that were performed to compare the effect of the joint angle estimation model type on each of the performance metrics. Each of the error metrics (Figure 6A) was found to differ significantly between model types, as the RF joint angle estimation model was found to have performed with a significantly lower model error (${RMSE}_{RF,model}$ = 4.14 ± 0.82°), realized error (${RMSE}_{RF,realized}$ = 5.63 ± 1.10°), and no-lag error (${RMSE}_{RF,nolag}$= 5.39 ± 0.88°) when compared with the kinematically governed model (${RMSE}_{K,model}$ = 8.18 ± 2.11°; ${RMSE}_{K,realized}$ = 6.77 ± 1.27°; ${RMSE}_{K,nolag}$= 6.43 ± 0.94°). Conversely, the RF model only performed with a significantly lower actuation time ($t_{RF,actuation}$ = 116 ± 17.1 ms) when compared with the kinematically governed model ($t_{K,actuation}$ = 131 ± 11.9 ms), while model type was found to have no significant effect on the phase delay of the exoskeleton mock-up system ($t_{RF,phase}$ = 21.0 ± 20.8 ms; $t_{K,phase}$ = 27.0 ± 24.1 ms).

### 3.2. Visual Inspection of Exoskeleton Mock-Up Testbed Performance

**Figure 7:**
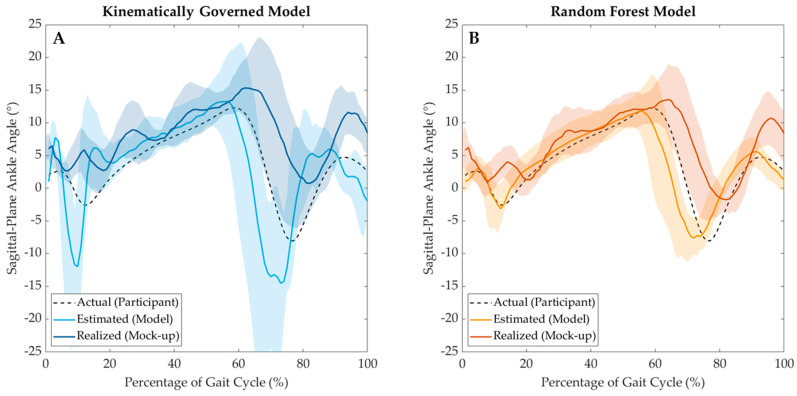
Visual representation of the average estimated ($\hat{\theta }$) and realized ($\theta_{mockup}$) testbed joint angles for the (**A**) kinematically governed extrapolation model and (**B**) RF model. Each estimated and realized curve is overlaid on top of the averaged actual participant ankle angle curve ($\theta_{actual}$) as a function of the gait cycle percentile.

In addition to evaluating the defined performance metrics, the average estimated joint angles ($\hat{\theta }$) and average realized mock-up angles ($\theta_{mockup}$) were plotted against the average actual joint angles during data collection ($\theta_{actual}$) at each percentage of the gait cycle to provide a visual representation of how the exoskeleton mock-up was influenced by each of the two joint angle estimation models. As depicted in Figure 7, the error between the $\theta_{mockup}$ curve and the $\theta_{actual}$ curve visually represents the ${RMSE}_{realized}$ error metric, while the temporal shift between the $\hat{\theta }$ and $\theta_{mockup}$ curves and between the $\theta_{actual}$ and $\theta_{mockup}$ curves visually represents the $t_{actuation}$ and $t_{phase}$ temporal metrics, respectively.

Note that Figure 7 does not visually illustrate the ${RMSE}_{model}$ performance metric. Instead, the estimated joint angles are aligned temporally with when they were calcualted, *not* aligned temporally at the estimation horizon alongside the actual joint angles they were estimated to represent. As such, both Figure 7A and Figure 7B clearly depict $\hat{\theta }$ curves that precede the $\theta_{actual}$ curves, simply because the $\hat{\theta }$ curves were estimated $t_{hzn}=$ 100 ms before the $\theta_{actual}$ curves.

**Figure 8:**
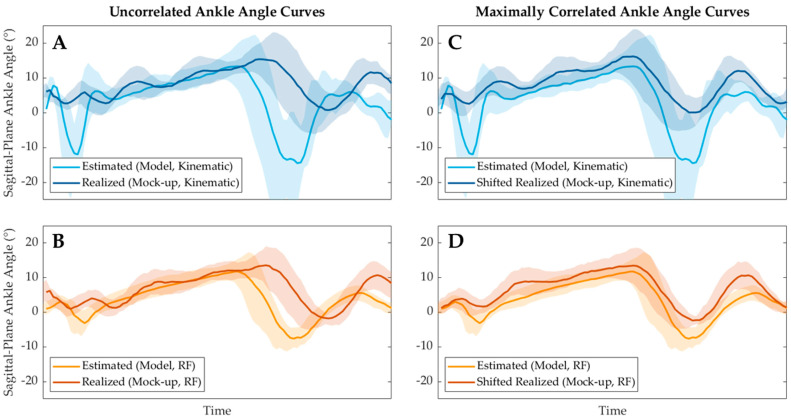
Representation of the cross-correlation technique used to characterize the $t_{phase}$ and ${RMSE}_{nolag}$ performance metrics. The estimated and actual ankle angle curves of each model (**A**,**B**) were shifted incrementally to maximize the cross-correlation between the curves (**C**,**D**). The temporal difference between the uncorrelated curves and the maximally correlated curves of each model (i.e., how much the two curves needed to be shifted to maximally correlate) represents the $t_{phase}$ metric, while the root-mean-square error between the now maximally correlated curves represents the ${RMSE}_{nolag}$ metric.

Figure 8 illustrates the results of the cross-correlation technique used to characterize the $t_{phase}$ and ${RMSE}_{nolag}$ performance metrics. Figure 8A,B illustrate the uncorrelated $\hat{\theta }$ and $\theta_{actual}\;$curves of each model, as shown prior in Figure 7. However, Figure 8C,D show the same curves after they have shifted to produce the maximum cross-correlation of the $\hat{\theta }$ and $\theta_{actual}\;$curves. This necessary temporal shift to maximally cross-correlate and the remaining error between the $\hat{\theta }$ and $\theta_{actual}\;$curves after shifting characterize the $t_{phase}$ and ${RMSE}_{nolag}$ performance metrics, respectively. The $t_{actuation}$ metric was characterized similarly to the process depicted in Figure 8, shifting to induce maximum cross-correlation between the $\hat{\theta }$ and $\theta_{mockup}$ curves instead.

## 4. Discussion

### 4.1. Results and Hypotheses Discussion

This study sought to establish and deploy a modular approach to testing the physical manifestation characteristics of joint angle estimation models on an exoskeleton mock-up testbed. This modular approach provides a methodological framework for future studies so that the physical manifestation characteristics of an exoskeleton testbed can be analyzed as a function of specific sensor configurations, joint angle estimation models, exoskeleton mock-up designs, or controller architectures. Modularizing this test configuration assists in analyzing the effects of a single design parameter by mitigating the variability in the other modules. As the field of off-board, non-intervention exoskeleton mock-up testing begins to grow, this approach seeks to promote the uniformity of testing conditions and performance quantification.

To test the viability of this modular approach, two different joint angle estimation model modules (a kinematically governed model and an RF model) were deployed alongside otherwise identical modules. The primary hypothesis of this study posited that the RF model would demonstrate a lower realized error between the exoskeleton mock-up and the actual human kinematics than the kinematic extrapolation model would. This hypothesis was confirmed by analyzing several error measures, specifically the realized error and the no-lag error performance metrics, as the RF model performed with both a significantly lower ${RMSE}_{realized}$ and ${RMSE}_{nolag}$ than the kinematically governed model (Figure 6A). Confirmation of this hypothesis indicates that, at least for this test configuration, a lower model error (as shown by the ${RMSE}_{model}$ results of this study and by the offline analysis performed in [30]) correlates to a lower realized error on a physical mock-up system. Interestingly, it should be noted that, even after temporally aligning the estimated and actual joint angle curves and thereby mitigating the positional error due to temporal differences, the ${RMSE}_{nolag}$ was not substantially lower than the ${RMSE}_{realized}$ of each model (Figure 6A). This indicates that the realized positional error of each model, ${RMSE}_{realized}$, can be attributed to an inability to follow the actual kinematic trajectory of the participants more so than an inability to actuate at a proper time. This finding is important for this testbed configuration because it prioritizes an immediate need to decrease model error or improve the control law, even before reducing delays associated with the actuation system.

The second hypothesis of this study stated that the joint angles estimated by the kinematic extrapolation model would be physically realized more quickly on the exoskeleton mock-up testbed than joint angles estimated by the RF model. However, this hypothesis was rejected when comparing testbed actuation times, as $t_{K,actuation}$ was shown to be significantly larger than $t_{RF,actuation}$ (Figure 6B). This is likely because the kinematically governed extrapolation model’s estimations are highly erroneous with rapid changes in direction (as is evident at the local minimums and maximums in the ankle angle plots in Figure 7A). Because these kinematically governed estimations are substantially more erroneous than the RF estimations at these extremes, the exoskeleton mock-up must actuate for longer to try to reach these desired angular positions. Subsequently, the $t_{phase}$ performance metric was calculated to explain whether this increase in actuation time was meaningful for an exoskeleton application. Even though the $t_{actuation}$ metric varied significantly between model types, no significance was found between $t_{K,\;phase}$ and $t_{RF,\;phase}$ (Figure 6B). This finding likely indicates that the longer $t_{K,actuation}$ was effectively mitigated by the kinematically governed model calculating joint angle estimations faster than the RF model (reported as the model runtime in [30]). Effectively, for this testbed configuration, these two sequential processes (estimating a future joint angle and subsequently actuating to it) were found to not vary significantly between model types. Even still, the exoskeleton mock-up lagged behind the actual participant gait trajectories when informed by both the kinematic extrapolation and RF models. This temporal lag indicates that the exoskeleton mock-up was unable to actuate quickly enough to precede the underlying human motion even though the joint angle estimation models were estimating future angles at $t_{hzn}$ = 100 ms into the future. This finding, in conjunction with the finding from the first hypothesis, emphasizes the need to expand the methodology of this study to longer estimation horizons ($t_{hzn}$ > 100 ms) while improving single-sensor estimation model performance at these horizons in order to (1) follow the trajectory of the underlying human kinematics less erroneously while simultaneously (2) allowing sufficient time for the exoskeleton mock-up to actuate to prevent the system from lagging behind the operator’s intended motion. Achieving improved model performance at larger estimation horizons would provide the exoskeleton mock-up with additional time to actuate, thus potentially preventing the mock-up from lagging behind the operator’s intended kinematics.

Reducing model error at longer estimation horizons is likely most achievable by altering the sensor configuration and model type. However, one of the primary aims of this study was to analyze the performance of an exoskeleton testbed informed by joint angle estimation models using only a single sensor configuration. So, while modifying this modular testbed system to include a larger sensor array may improve performance and will likely be the primary focus of several future studies, the current, single-sensor configuration serves a valuable role as a baseline comparator for more complex future testbeds. Even still, this single-sensor system may be sufficient for some gait assistance applications. A primary concern with this current exoskeleton mock-up and its associated joint angle estimation models was the erroneous joint angles realized by the controlled testbed, with root-mean-squared errors in the range of 6.77 ± 1.27° and 5.63 ± 1.10° for the kinematic extrapolation and RF models, respectively. However, Fournier Belley et al. (2016) [38] demonstrated that operators of robotic ankle orthotics were only able to detect robotic movement errors at a threshold of 5.31 ± 2.12°. Therefore, an exoskeleton mock-up informed by one of these single-sensor joint angle estimation models would likely perform near the boundary of human error perception. Therefore, exploring the effects of a more mature exoskeleton prototype controlled via a single sensor on an operator may help answer additional questions about the joint angle estimation models’ performance, such as “how accurate is accurate enough?”

### 4.2. Limitations and Future Work

To the knowledge of the authors, this was the first study that explored the physical manifestation performance of joint angle estimation models informed by a single sensor. Because of this, however, several limitations to this study need to be addressed. First and foremost are the nonrepresentational boundary conditions of this study. While an exoskeleton mock-up is beneficial for testing the physical manifestation response of joint angle estimation models without the need for human subject intervention, the mock-up testbed does not fully represent the operator/exoskeleton complex. Most significantly, antagonistic muscle contraction, lower limb inertia, ground reaction forces, and operator/exoskeleton interaction forces are not present in this mock-up testbed. Not including these boundary conditions in the mock-up may mitigate extrapolating these results to an online exoskeleton prototype application. Modeling these boundary conditions in a future iteration of the exoskeleton mechanics module would allow the mock-up to better represent deployment on a human operator. However, even without accounting for these boundary conditions, the exoskeleton mock-up configuration is still capable of quickly providing valuable information about a system’s temporal and error performance metrics that can be used in rapid exoskeleton prototyping.

Several limitations arose regarding the mechanical and control architecture of this testbed system, including both the actuation technique and the control law. While most exoskeleton prototypes only provide plantarflexion assistance [39], many of them deploy a motor on the joint of interest to provide direct torque assistance, rather than providing indirect torque assistance by linearly actuating a moment arm. The linear actuation technique deployed in this study, alongside the passively dorsiflexing elastic band, may have contributed to the inaccuracy of the realized joint angles, as providing actuation through several consecutive components allows feedback error to compound when trying to control the system. Likewise, later iterations of this testbed should seek to optimize the mock-up’s joint-rotation sensor, as the encoder used in this study was selected based on prior exoskeleton manufacturing literature [34] and not because of a comparative, quantitative analysis of available sensor systems. Additionally, a simple, proportional velocity controller was deployed on this testbed because of the previously unknown behavior of the exoskeleton mock-up’s dynamics. This naïve controller design must be optimized in future studies to mitigate the undesirable oscillatory actuation of the mock-up, as is evident in Figure 7. Although these results serve as a baseline for future controller designs, future studies should incorporate the recorded testbed dynamics into the controller’s architecture to further decrease the realized error of the system.

Additionally, extending these joint angle estimation models to different actions and populations may pose some limitations. As discussed briefly in Pollard et al. (2024), the simple analytical-based estimation models presented in this study may yield higher errors if used in applications where rapid changes in joint angle are present (such as running or changes in direction) because they are inherently extrapolative in nature (i.e., they assume that rapid changes in direction will *not* occur over the estimation horizon). As such, additional work needs to be performed to characterize these analytical models’ performance under different actions to understand their viability outside of level-ground walking applications. The training regime of the RF models may also pose limitations as each model was developed on a subject-specific basis, thereby limiting the models’ generalizability to a general population. While this subject-specific training regime may not be limiting for military or rehabilitative applications that are tailor-designed for a specific operator, evaluating the performance of subject-generic models for generalizable applications should be explored in future studies using this methodological framework.

Finally, although the averaged estimated and realized joint angle curves presented in Figure 7 and Figure 8 are smooth upon visual inspection, each individual trial was relatively noisy compared to these averaged curves. As such, the maximum correlation method used to characterize the actuation and phase delays of the system was identified as a potential limitation. This method has been commonly deployed to estimate the temporal lag of non-uniform curves. However, as implemented in this study, if the estimated or realized joint angles during a single gait cycle became highly erroneous and noisy, maximally correlating these two curves to characterize $t_{actuate}$ (or correlating the actual and realized curves to characterize $t_{phase}$) may yield meaningless results, as maximally correlating two curves with low signal-to-noise ratios may improperly align the signals. While visual inspection of the correlation shifts encouraged the authors that this limitation did not significantly impact the results of this study, this method should be noted as a potential limitation for future studies that may seek to characterize the actuation and phase delays of a system with substantially noisy results. Future studies can mitigate this potential limitation by deploying more accurate joint angle estimation models or better-performing control algorithms that yield realized joint angle curves that more closely match the kinematics of the operator.

## 5. Conclusions

This study successfully demonstrated the feasibility of implementing a modularized approach to joint angle estimation and exoskeleton mock-up testing. The exoskeleton mock-up was developed to serve a role in the rapid testing of estimation models, sensor arrays, and control algorithms and in the rapid prototyping of exoskeleton mechanics and actuation techniques. The results of testing kinematically governed and RF estimation models, which were informed by a single potentiometer at the ankle, indicated that the RF model physically manifested its estimations more accurately and more quickly than the kinematically governed extrapolation model on a physical exoskeleton mock-up. Although it remains unknown if these single-sensor models performed accurately enough to be deployed on a more mature exoskeleton prototype (that could be worn by human subjects), the simplicity of the exoskeleton mock-up testbed explored in this study allows these results to serve as a foundational baseline for subsequent studies that will seek to increase the joint angle estimation models’ accuracy, improve the controllability of the mock-up, and decrease the actuation delays of the system.

## Data Availability

The data presented in this study are available on request from the corresponding author. The data are not publicly available due to the human-subject-derived nature of the dataset.
